# IVS1 −397T**>**C Estrogen Receptor ****α**** Polymorphism Is Associated with Low-Grade Systemic Inflammatory Response in Type 1 Diabetic Girls

**DOI:** 10.1155/2014/839585

**Published:** 2014-01-02

**Authors:** Monika Ryba-Stanisławowska, Karolina Rybarczyk-Kapturska, Agnieszka Brandt, Małgorzata Myśliwiec, Jolanta Myśliwska

**Affiliations:** ^1^Department of Immunology, Medical University of Gdańsk, Dębinki 1, 80-211 Gdańsk, Poland; ^2^Clinic of Pediatrics, Department of Diabetology and Endocrinology, Medical University of Gdańsk, 80-211 Gdańsk, Poland

## Abstract

*Purpose*. The study aimed to investigate the influence of estrogen receptor **α** (ER-**α**) genotypes on inflammatory response and development of microvascular complications in girls with type 1 diabetes. *Methods*. 152 young regularly menstruating girls with diagnosed type 1 diabetes and 84 young, healthy menstruating girls were recruited. ER-**α** genotyping was carried out by PCR. Serum concentrations of 17**β**-estradiol, as well as IL-6, TNF-**α**, VEGF, and IL-10, were measured. CD4^+^Foxp3^+^ TH17 cells were isolated and analyzed by flow cytometry. *Results*. Type 1 diabetic girls carrying TT genotype were characterized by the lowest serum estradiol level and IL-10 and highest IL-6, TNF-**α** , and VEGF. The association between the level of certain cytokine and the genetic variant of estrogen receptor **α** polymorphism was analyzed. Frequencies of CD4^+^Foxp3^+^ TH17 cells were also enhanced in TT bearing girls with type 1 diabetes and correlated with the level of analyzed cytokines. In addition, the correlation between serum estradiol level and cytokine concentrations was observed. *Conclusions*. We propose that TT variant of estrogen receptor **α** polymorphism may be associated with enhanced inflammatory response, which in turn may lead to acceleration of diabetic retino- and nephropathy in girls with type 1 diabetes. This finding may help the physicians to predict the onset and progression of diabetic microvascular complications.

## 1. Introduction

One of the most analyzed genetic factors that control autoimmunity is polymorphism of certain genes, which in case of particular alleles contributes to the protection against some autoimmune diseases. Conversely, however, some genetic variants induce the development and the progression of such illnesses [1].

Another factor that affects autoimmunity is gender, and so females are thought to be more susceptible to develop autoimmune diseases [2–5].

The prevalence of autoimmune diseases in females may depend in part on the influence of sex hormones on the immune system [3, 6]. It is well known that the autoimmune response in some diseases is hampered during the pregnancy, when the levels of estrogens are high [3, 6]. Estrogens are able to induce the expansion of suppressor regulatory T cells (Tregs) [7–9], which makes them potentially protective factors in the development of autoimmune diseases. The function of Tregs was shown by us and others to be compromised in type 1 diabetic subjects [10–13]. Furthermore we have found that the level of Tregs, as well as their ability to express Foxp3, may depend in part on estrogen receptor *α* polymorphism, which may simultaneously influence the inflammatory response in DM1 (diabetes mellitus type 1) females [14].

Chronic low-grade inflammation related to type 1 diabetes is manifested by detectable levels of serum biomarkers of inflammation and may contribute to the development of late diabetic microvascular complications: retino- and nephropathy [15, 16]. There is data showing that DM1 patients with poor metabolic control have higher CRP levels and produce more proinflammatory cytokines [12, 16]. Among various cytokines involved in promoting and maintaining chronic inflammatory response, TNF-*α* (tumor necrosis factor-*α*) and IL-6 are systemically increased in patients with type 1 diabetes [16–19]. TNF-*α* and IL-6, in turn, have potential to upregulate the expression of vascular endothelial growth factor (VEGF), which induces neovascularization during retino- and nephropathy progression [19–21]. Moreover, TNF-*α* and IL-6 were also shown by us and others to have impact on regulatory conditions and Treg/Th17 balance in type 1 diabetic patients [11, 22, 23]. Th17 cells are involved in the pathogenesis of inflammatory and autoimmune diseases and they also predominate in patients with type 1 diabetes [24–27].

Taking all these into account, we aimed to examine if the IVSI −397T>C estrogen receptor *α* polymorphism is associated with chronic inflammatory response and microvascular complications in girls with type 1 diabetes.

## 2. Methods

### 2.1. Subjects

The study group consisted of 152 young, regularly menstruating girls with diagnosed type 1 diabetes who were recruited from the Clinic of Pediatrics, Department of Diabetology and Endocrinology, Medical University of Gdańsk. Mean age of patients was 14 ± 3.7 years. Type 1 diabetes was defined according to the criteria of the American Diabetes Association [28]. Patients with coexisting autoimmune, chronic, and acute, inflammatory diseases were excluded from the study. In all examined patients the C-peptide levels were below 0.5 ng/mL. All patients were treated with humanized insulin at doses of 0.87 ± 0.2 mg/kg. At the time of sampling blood glucose level along with biochemical measurement of renal function, lipid status, C-reactive protein (CRP) and glycosylated hemoglobin (HbA1c) was monitored.

The control group consisted of 84 young, healthy menstruating girls aged 14.5 ± 5.7 years recruited during control visits in an outpatient clinic. No signs of autoimmune, chronic, inflammatory, or neoplastic disease at the time of sampling and no evidence of DM1 in their families were disclosed as confirmed by medical records, laboratory examination, and laboratory tests.

The blood from all girls was collected in the follicular phase (between days two and four) of menstrual cycle. Additionally, the level of plasma 17*β*-estradiol was determined in all individuals. All subjects gave informed consent and the study followed the principles of the Declaration of Helsinki and was approved by the Ethics Committee of the Medical University of Gdańsk.

### 2.2. Single Nucleotide Polymorphism

Genomic DNA from all the subjects was isolated from EDTA-stabilized blood using the Blood Mini Isolation Kit (A&A Biotechnology, Poland). Polymorphism of the *ER-*α** gene was analyzed by polymerase chain reaction-restriction fragment length polymorphism (PCR-RFLP). The DNA amplification was performed with 5′-CAACCAAGACTACAAGTAC-CGCGTCAGTGA-3′ oligonucleotide as forward primer and with 5′-AACCAGCGGAAGAGGTCAAGGG-3′ oligonucleotide as reverse primer. The amplification product (1374 base pairs) was incubated with 2.5 U of the restriction enzyme Pvu II (MBI Fermentas, Inc., USA) for 16 hours in 37°C. The allele size was as follows: T: 936 + 438, C: 1374 kb. The DNA restriction fragments were visualized under UV light on 2% agarose gel with ethidium bromide staining.

### 2.3. Isolation of Th17 Cells

Heparinized venous blood samples were collected and used to isolate PBMCs (peripheral blood mononuclear cells).

PBMCs were separated by density gradient preparation over Ficoll-Uropoline. To analyze Th17 cells, PBMCs were suspended at a density of 2 × 10^6^ cells/mL and cultured in RPMI 1640 supplemented with 5% heat-inactivated fetal calf serum (FCS). Cultures were stimulated with 50 ng/mL of phorbol myristate acetate (PMA) (Sigma, USA) plus 1 *μ*L/mL of ionomycin (Sigma, USA) for 4 h in the presence of 1 *μ*L/mL of monensin (BioLegend, USA).

### 2.4. Flow Cytometric Staining and Analysis

Cells were stained with anti-CD4 antibody (IgG1, *κ* mouse Pe/Cy5, Clone RPA-T4, BioLegend, USA) and incubated for 20 minutes at room temperature. Then intracellular staining for the expression of IL17A with anti-IL17A (IgG1, *κ* mouse FITC, Clone BL168, BioLegend, USA) antibody was performed. Expression of cell surface and intracellular markers were assessed using flow cytometry (LSRII, Becton Dickinson, USA) after gating on live cells determined by scatter characteristics. Positive signal for each staining was established using appropriate isotype control. Data were analyzed by FACSDiva 6.0 Software (Becton Dickinson, USA).

### 2.5. Cytokine Measurements

Plasma cytokines were measured at the time of inclusion in the study. Serum levels of IL-6, tumor necrosis factor-*α* (TNF-*α*), vascular endothelial factor (VEGF), and IL-10 were determined using commercial enzyme-linked immunosorbent assay kits (R&D Systems, Minneapolis, Minn., USA) according to the manufacturer's protocol.

### 2.6. Statistical Analyses

The results were analyzed using Statistica, ver. 9.0 (StatSoft, Inc., USA). Conformation of the allele frequencies to the Hardy-Weinberg equilibrium proportions was tested by the *χ* ^2^ test. Normally distributed variables were analyzed with the one-way ANOVA test. The post hoc NIR test was applied to asses statistical significance. For comparison of the skew distributed variables the nonparametric Kruskal-Wallis ANOVA test was applied. In addition, the multiple regression analysis was used to discover the relationships among variables. The level of significance was set at *P* ≤ 0.05.

## 3. Results

### 3.1. The IVS1 −397T>C Estrogen Receptor *α* Polymorphism and Clinical Characteristics of Patients

The characteristics of type 1 diabetic girls enrolled in the study are presented in Table 1. The genotype frequencies in DM1 group were as follows: CC, 24.3%, CT, 46.1%, TT, 29.6% and conformed to the Hardy-Weinberg equilibrium (*P* = 0.54). The genotype frequencies in the group of healthy girls were CC, 21.4%, CT, 55.9%, and TT, 22.6%. The genotype distributions were not statistically different between the DM1 patients and healthy group (*P* = 0.42; *χ* ^2^ test). Clinical features of the patients differing in the IVS1 −397T>C estrogen receptor *α* polymorphism were similar with respect to age, duration of diabetes, HbA1c, BMI, albumin excretion rate, and serum creatinine level. However, we observed that girls bearing CC genotype had lower BMI and CRP level in comparison to TT bearing patients (*P* = 0.01 and *P* = 0.02, resp.).

As to estrogen concentrations, the level of 17*β*-estradiol was measured in the serum of all examined girls between days two and four of the menstrual cycle. In the healthy, control group the level of estradiol did not differ between CC, CT and TT variants of *ER-*α** gene (Table 2, *P* = 0.8). In DM1 group, however, girls carrying CC genotype were characterized by the highest estradiol level. The level of this hormone was decreasing along with the presence of the T allele copies. To confirm this observation, we performed the multiple regression analysis, which revealed that the presence of −397T allele (*β* = [−0.36]) had a significant effect (*P* = 0.005) on serum level of 17*β*-estradiol. Patient's age (*β* = 0.07; *P* = 0.63), as well as the duration of diabetes (*β* = 0.19; *P* = 0.2), had no effect on estradiol serum level.

Moreover, the group of girls with type 1 diabetes was analyzed with regard to existing microvascular complications: retino- and nephropathy. The results are shown in Table 3. The TT genotype was more common in DM1 girls with nephropathy in comparison to nephropathy-free DM1 subjects (Table 3(a)). Similarly, TT variant was more common in DM1 girls with retinopathy than in DM1 girls without this complication (Table 3(b)).

### 3.2. Th17 Cell Frequencies in Type 1 Diabetic Girls according to Genetic Variant of Estrogen Receptor *α* Polymorphism

As it was mentioned, our previous studies showed the association between the −397T>C polymorphism of the estrogen receptor *α* gene and the quantitative characteristics of regulatory CD4^+^Foxp3^+^ T cells in girls with type 1 diabetes [14]. Taking into account the fact that in type 1 diabetic patients the balance between regulatory T cells and their opposites, Th17, is disrupted [22, 26, 27], we have decided to check if and how the frequency of the latter cells depends on the −397T>C estrogen receptor *α* polymorphism. When analyzing Th17 cell frequencies according to genetic variant of estrogen receptor *α* polymorphism, we found differences between these cells but only in the group of girls with type 1 diabetes (Table 4). The post hoc NIR test revealed the significant difference between CC and TT carriers (*P* = 0.05).

### 3.3. Serum Levels of Analyzed Cytokines according to Genetic Variant of Estrogen Receptor *α* Polymorphism

Chronic low-grade inflammation is involved in development and progression of diabetic microvascular complications: retino- and nephropathy. Therefore, we aimed to analyze the levels of cytokines, IL-6, TNF-*α*, VEGF, and IL-10, in serum of girls with type 1 diabetes and find out whether estrogen receptor *α* polymorphism has relevance for their production.

The statistical analysis showed that DM1 girls with coexisting microvascular complications had higher levels of IL-6 (*P* < 0.0001), TNF-*α* (*P* < 0.0001) and, VEGF (*P* = 0.05) than their healthy counterparts from the control group (Table 5). In addition, the levels of these cytokines were higher in DM1 girls with coexisting complications in comparison to DM1 girls free from retino- and nephropathy. As to the serum level of IL-10, DM1 girls with coexisting complications produced less of this cytokine than girls from two other groups (Table 5).

**Figure 1:**
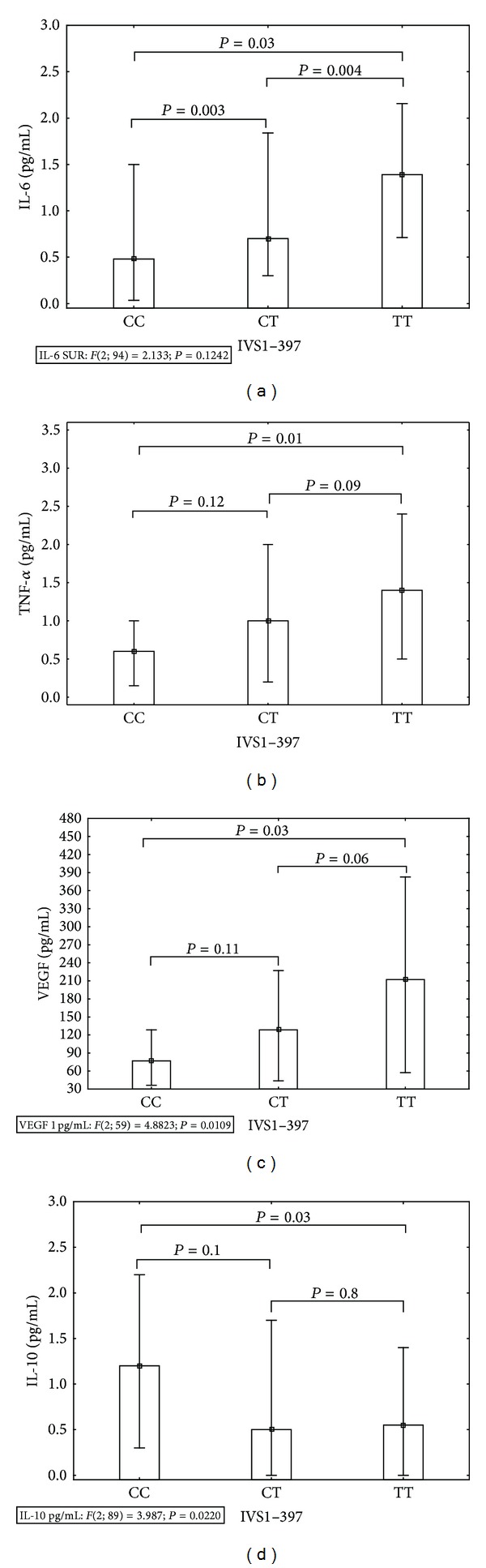
Serum levels of IL-6, TNF-*α*, VEGF, and IL-10 in DM1 girls differing in the IVS1 **−**397T>C polymorphism. In DM1 group the value of serum cytokines was measured by ELISA and analyzed according to different genetic variants of the IVS1 –397T>C estrogen receptor *α* polymorphism. (a) The mean value (25/75 percentiles) of IL-6 was 0.48 (0.03/1.5), 0.7 (0.3/1.84), and 1.39 (0.7/2.157) pg/mL for CC, CT, and TT, respectively. (b) The mean value (25/75 percentiles) of TNF-*α* was 0.6 (0.15/1), 1 (0.2/2), and 1.4 (0.5/2.4) pg/mL for CC, CT, and TT, respectively. (c) The mean value (25/75 percentiles) of VEGF was 76.89 (36.28/128.39), 128.45 (43.89/227.04), and 221.1 (57.4/382.76) pg/mL for CC, CT, and TT, respectively. (d) The mean value (25/75 percentiles) of IL-10 was 1.2 (0.3/2.2), 0.6 (0.0/1.7), and 0.5 (0.0/1.1) pg/mL for CC, CT, and TT, respectively. Differences were calculated by the Kruskal-Wallis ANOVA test and the post hoc NIR test.

In the next step of our work, we analyzed the association between the level of certain cytokine and the genetic variant of estrogen receptor *α* polymorphism. The results are shown in Figure 1. We found that type 1 diabetic girls bearing TT genotype produced higher levels of IL-6, TNF-*α*, and VEGF compared to their CC and CT counterparts. The level of these cytokines was decreasing along with the presence of the C allele copies. Using multiple regression analysis, we confirmed that the presence of −397T allele is associated with an increase in production of IL-6 (*β* = 0.24; *P* = 0.03), TNF-*α* (*β* = 0.42; *P* = 0.04), and VEGF (*β* = 0.25; *P* = 0.04). In contrast, TT genotype bearing girls produced less IL-10 than their CC and CT counterparts (Figure 1). Multiple regression analysis showed that the presence of −397T allele significantly decreases the level of IL-10 (*β* = [−0.27]; *P* = 0.01). In case of all analyzed cytokines the post hoc analysis revealed the significant difference between CC and TT carriers (*P* ≤ 0.03).

### 3.4. The Association of Th17 Cell Frequencies with Analyzed Cytokines

To extend our research in this area, we looked at the potential relationship between the concentration of analyzed cytokines, the level of serum 17*β*-estradiol, and the frequency of CD4^+^IL17A^+^ Th17 cells in girls with type 1 diabetes. We found an inverse correlation between 17*β*-estradiol level and concentrations of IL-6, TNF-*α*, and VEGF (Table 6). In addition, the frequency of Th17 cells was positively correlated with the levels of these three inflammatory cytokines (Table 6). As to the level of IL-10, we detected a positive correlation between its concentration and serum 17*β*-estradiol as well as Th17 frequencies (Table 6).

## 4. Discussion 

The increased incidence of autoimmune disorders among female patients gave rise to the great interest in the regulation of the immune response by sex hormones. In the present paper we analyzed the association of the estrogen receptor *α* polymorphism with inflammatory response in regularly menstruating girls with type 1 diabetes. Similarly to our previous paper regarding IVS1 −397T>C polymorphism [14], we found that TT genotype is associated with enhanced inflammatory response. TT genotype bearing girls produced less estradiol and more CRP than their CT and CC genotype bearing counterparts. This is consistent with papers regarding IVS1 −397T>C ER-*α* polymorphism in women with coronary heart disease [29–31]. Studies of Herrington et al. [30, 31] showed that the CC genotype carriers may bind estrogen much more strongly than TT bearing individuals. It is because the C allele, but not the T allele, contains a functional binding site for the transcription factor B-myb, which may upregulate the transcription of *ER-*α** gene [30, 31]. Decreased gene transcription resulting in reduced expression of *α* estrogen receptors may cause estrogen signaling to be less effective and thus attenuate its anti-inflammatory effect. This is consistent with studies suggesting that lower estrogen concentration is associated with increased production of proinflammatory cytokines [32–34]. TT bearing DM1 girls enrolled in the present study produced higher levels of proinflammatory cytokines (TNF-*α*, IL-6, and VEGF) than CT and CC carriers. Moreover, the production of these cytokines was negatively correlated with serum estradiol level. IL-6 and TNF-*α* are activators of NF-*κ*B (nuclear factor-*κ*B) pathway, which is an important contributor to the pathogenesis of microvascular complications [35]. In addition, they are capable of upregulating VEGF expression [19–21, 36], which was shown to play a role in pathogenesis of retino- and nephropathy [21, 37]. Conversely, VEGF is able to induce the production of IL-6 and TNF-*α* [38]. This continuous activity of proinflammatory molecules initiates a positive feedback loop resulting in development and subsequent progression of microangiopathies [15–19]. Indeed, in our study DM1 girls with retino- or nephropathy were more common among patients with genotype TT than among those with genotypes CT/CC. We can speculate that T allele is responsible for more severe inflammatory response leading to more rapid progression of diabetic complications. Inflammation related to TT variant of the IVS1 −397T>C estrogen receptor *α* polymorphism is even more likely, because DM1 girls bearing TT genotype produced less anti-inflammatory cytokine IL-10 than CT and CC carriers. This is consistent with studies done by Verthelyi, who showed that the level of IL-10 producing cells increases under the influence of estrogens [39], and DM1 girls bearing TT genotype produced this hormone at the lowest level.

Another important observation from the current study is the fact, that type 1 diabetic girls carrying TT genotype had higher frequency of CD4^+^IL17A^+^ T cells. Activation of Th17 cells is thought to be involved in autoimmune and inflammatory pathologies [24]. In our previous paper we showed the prevalence of Th17 cells in patients with type 1 diabetes. The study was done on a group of 32 male and female patients and showed the inverse correlation between the level of Th17 cells and regulatory CD4^+^IL17A^+^ Tregs [27]. The results presented in a current study confirm and expand those previously reported. Furthermore, the observation that higher frequency of Th17 cells is seen in TT carriers extends the work of the previous paper [14], in which we showed that TT genotype bearing DM1 girls had significantly decreased level of CD4^+^Foxp3^+^ Tregs.

To further confirm the proinflammatory effect of Th17 in DM1 subjects, we found positive correlation between frequency of these cells and the level of IL-6, TNF-*α* and VEGF. Interestingly, these three inflammation promoting cytokines were shown to be connected with Th17 cells. IL-6, while being associated with progression of diabetic complications [19, 40], also favors the differentiation of Th17 cells [23]. TNF-*α* is the cytokine that may have negative impact on regulatory T cells, hence they are not efficient at controlling the action of inflammatory cells [11, 41]. The greater the production of TNF-*α* is, the more likely the Treg/Th17 balance is shifted towards Th17. Moreover, Th17 cells are capable to produce TNF-*α* directly or in an indirect way [42]. In case of VEGF there is also data showing that the frequency of Th17 cells correlates with the level of this cytokine in patients with another autoimmune, inflammatory disease—systemic lupus erythematosus (SLE) [43]. Finally, studies done by Chung et al. showed that neutralizing antibody to IL17A significantly reduced the amounts of VEGF in tumor-bearing mice [44].

In conclusion, elevated levels of analyzed proinflammatory factors (Th17 cells, cytokines) in DM1 girls carrying TT genotype promote enhanced inflammatory response, which leads toward development and progression of diabetic complications. The TT genotype of IVS1 −397T>C estrogen receptor *α* polymorphism in type 1 diabetic girls may be associated with chronic inflammatory response manifested by the higher incidence of diabetic complications. This finding may help the physicians to predict the onset and progression of diabetic retino- and nephropathy.

## Figures and Tables

**Table 1:** Clinical characteristics of girls with type 1 diabetes differing in the IVS1 −397T>C estrogen receptor *α* polymorphism.

Clinical parameter	CC	CT	TT	*P*	P*	P**	P***
*N*	37	70	45				
Age (years)	14.2 ± 3.4	14.0 ± 3.6	13.2 ± 3.8	0.81	0.86	0.79	0.74
Duration of diabetes (years)	5.3 ± 3.3	4.1 ± 3.1	4.6 ± 3.8	0.53	0.09	0.58	0.36
BMI (kg/m^2^)	20.1 ± 3.5	19.4 ± 3.6	18.7 ± 3.8	0.05	0.28	0.39	**0.01**
HbA1c (%)	8.9 ± 2.5	8.6 ± 2.1	9.2 ± 2.4	0.21	0.28	0.17	0.67
CRP (mg/mL)	1.6 ± 1.4	1.8 ± 1.6	2.5 ± 2.3	0.1	0.5	0.06	**0.02**
Albumin excretion rate (mg/24 h)	19.8 ± 16.2	20.8 ± 19.4	23.3 ± 22.6	0.6	0.39	0.08	0.37
Serum creatinine level (mg/dL)	0.75 ± 0.13	0.74 ± 0.17	0.73 ± 0.16	0.98	0.33	0.34	0.61

Differences were calculated by the Kruskal-Wallis ANOVA test. Data are presented as mean ± SD.

*P*: the comparison between three analyzed genotypes CC, CT, and TT.

P*: post hoc comparison between patients bearing CC and CT genotypes.

P**: post hoc comparison between patients bearing CT and TT genotypes.

P***: post hoc comparison between patients bearing CC and TT genotypes.

**Table 2:** Serum level of 17*β*-estradiol in DM1 and healthy young girls differing in the IVS1 −397T>C estrogen receptor *α* polymorphism.

	CC	CT	TT	*P*	P*	P**	P***
Control group	217 ± 59	234.6 ± 152.9	220.1 ± 74.3	0.86	0.8	0.69	0.89
DM1	281.1 ± 262.6	248.3 ± 130.3	141.4 ± 75.5	**0.028**	0.28	0.06	**0.04**

Differences were calculated by the Kruskal-Wallis ANOVA test. Data are presented as mean ± SD.

The values are presented as pmol/L.

*P*: the comparison between three analyzed genotypes CC, CT and TT.

P*: post hoc comparison between patients bearing CC and CT genotypes.

P**: post hoc comparison between patients bearing CT and TT genotypes.

P***: post hoc comparison between patients bearing CC and TT genotypes.

**Table:** (a)

	Girls with DM1 *n* (%)	Girls with DM1 and nephropathy *n* (%)	Girls with DM1 without nephropathy *n* (%)
CC	37 (24.3)	4 (14.3)	33 (26.8)
CT	70 (46.1)	14 (50)	56 (45.5)
TT	45 (29.6)	10 (35.7)	34 (27.6)
*χ* ^2^ Pearson	—	2.07; *P* = 0.35	
*χ* ^2^ NW	—	2.25; *P* = 0.32	

Data were calculated with *χ* ^2^ Pearson's test.

*n*: number of patients.

**Table:** (b)

	Girls with DM1 *n* (%)	Girls with DM1 and retinopathy *n* (%)	Girls with DM1 without retinopathy *n* (%)
CC	37 (24.3)	2 (9.5)	35 (26.9)
CT	70 (46.1)	8 (38.1)	62 (47.7)
TT	45 (29.6)	11 (52.4)	33 (25.4)
*χ* ^2^ Pearson	—	7.11; *P* = 0.03	
*χ* ^2^ NW	—	6.99; *P* = 0.03	

Data were calculated with *χ* ^2^ Pearson's test.

*n*: number of patients.

**Table 4:** Flow cytometric analysis of CD4^+^IL17A^+^ T cells in girls differing in the IVS1 −397T>C estrogen receptor *α* polymorphism.

	CC	CT	TT	*P*	P*	P**	P***
CD4^+^IL17A^+^ T cells (%)							
Control group	1.1 (0.9/1.4)	0.9 (0.7/1.3)	1.2 (0.8/1.5)	0.4	0.42	0.29	0.68
DM1	1.5 (1.1/2.1)	2.1 (1.5/3.25)	2.69 (2.04/3.7)	0.18	0.09	0.95	**0.05**

PBMCs from type 1 diabetic (DM1) and healthy (control group) girls were cultured and stimulated as described in Section 2 and then stained with antibodies against CD4 and IL17A. The percentage of CD4^+^IL17A^+^ T cells was determined by flow cytometry. Analyzing CD4^+^IL17A^+^ cells, dot plots representing anti-CD4 versus SS were carried out to establish CD4^+^ and CD4^−^ lymphocyte gates. Then, the anti-CD4 versus IL17A from CD4^+^ gate dot plot was generated and the frequency of Th17 cells was determined.

Results are shown as median and 10./90. percentile. Differences were calculated by the Kruskal-Wallis ANOVA test.

*P*: the comparison between three analyzed genotypes CC, CT, and TT.

P*: post hoc comparison between patients bearing CC and CT genotypes.

P**: post hoc comparison between patients bearing CT and TT genotypes.

P***: post hoc comparison between patients bearing CC and TT genotypes.

**Table 5:** The level of serum cytokines in analyzed subjects.

	DM1 girls with coexisting microvascular complications (*n* = 41)	DM1 girls free from microvascular complications (*n* = 111)	Control group (*n* = 84)	*P*	P*	P**	P***
IL-6 (pg/mL)	0.9 (0.4/1.8)	0.8 (0.2/1.5)	0.4 (0.2/0.6)	**0.0004 **	0.34	**0.001 **	<**0.0001 **
TNF-*α* (pg/mL)	1.8 (1.1/2.8)	0.0 (0/0.8)	0.0 (0/0)	<**0.0001 **	<**0.0001 **	**0.007 **	<**0.0001 **
VEGF (pg/mL)	216.8 (57.4/439.3)	98.4 (43.6/181.1)	113.4 (29.6/289.7)	**0.03 **	**0.008 **	0.17	0.05
IL-10 (pg/mL)	0.5 (0/1.4)	0.6 (0.1/1.8)	0 (0/1.5)	0.44	0.14	**0.001 **	0.64

Data are presented as median and 10./90. percentile. Differences were calculated by the Kruskal-Wallis ANOVA test and post hoc NIR test.

*P*: comparison between three analyzed subject groups.

P*: post hoc comparison between DM1 girls with complications and DM1 group without complications.

P**: post hoc comparison between DM1 girls without complications and the control group.

P***: post hoc comparison between DM1 girls with complications and the control group.

**Table 6:** The results of the correlation analysis between serum level of 17*β*-estradiol, Th17 subset, and the concentrations of analyzed cytokines in DM1 patients.

	IL-6	TNF-*α*	VEGF	IL-10
Serum level of 17*β*-estradiol	*R* = [−0.2]	**R** = [−0.71]	**R** = [−0.86]	*R* = 0.2
	*P* > 0.05	*P* < 0.05	*P* < 0.05	*P* > 0.05
The percentage of CD4^+^IL17A^+^ T cells (%)*	**R** = 0.55	*R* = 0.45	**R** = 0.46	**R** = [−0.64]
	*P* < 0.05	*P* > 0.05	*P* < 0.05	*P* < 0.05

The Spearman test was used to calculate the strength of correlation.

*The percentage of cells among peripheral blood lymphocytes.

## Abbreviations

IL: Interleukin
DM1: Diabetes mellitus 1
Tregs: Regulatory T cells
TNF: Tumor necrosis factor
VEGF: Vascular endothelial growth factor.

## References

[1] Tang L, Wang L, Liao Q (2013). Genetic associations with diabetes: meta-analyses of 10 candidate polymorphisms. *PLoS One*.

[2] Quintero OL, Amador-Patarroyo MJ, Montoya-Ortiz G, Rojas-Villarraga A, Anaya J-M (2012). Autoimmune disease and gender: plausible mechanisms for the female predominance of autoimmunity. *Journal of Autoimmunity*.

[3] Rubtsov AV, Rubtsova K, Kappler JW, Marrack P (2010). Genetic and hormonal factors in female-biased autoimmunity. *Autoimmunity Reviews*.

[4] Nussinovitch U, Shoenfeld Y (2012). The role of gender and organ specific autoimmunity. *Autoimmunity Reviews*.

[5] Gleicher N, Barad DH (2007). Gender as risk factor for autoimmune diseases. *Journal of Autoimmunity*.

[6] González DA, Díaz BB, Rodríguez Pérez Mdel C, Hernández AG, Chico BN, de León AC (2010). Sex hormones and autoimmunity. *Immunology Letters*.

[7] Polanczyk MJ, Carson BD, Subramanian S (2004). Cutting edge: estrogen drives expiansion of the CD4^+^CD25 ^+^ regulatory T cell compartment. *The Journal of Immunology*.

[8] Tai P, Wang J, Jin H (2008). Induction of regulatory T cells by physiological level estrogen. *Journal of Cellular Physiology*.

[9] Arruvito L, Sanz M, Banham AH, Fainboim L (2007). Expansion of CD4^+^CD25^+^ and FOXP3^+^ regulatory T cells during the follicular phase of the menstrual cycle: implications for human reproduction. *The Journal of Immunology*.

[10] Lawson JM, Tremble J, Dayan C (2008). Increased resistance to CD4^+^CD25^hi^ regulatory T cell-mediated suppression in patients with type 1 diabetes. *Clinical and Experimental Immunology*.

[11] Ryba M, Marek N, Hak Ł (2011). Anti-TNF rescue CD4^+^Foxp3^+^ regulatory T cells in patients with type 1 diabetes from effects mediated by TNF. *Cytokine*.

[12] Ryba M, Rybarczyk-Kapturska K, Zorena K, Myśliwiec M, Myśliwska J (2011). Lower frequency of CD62L^high^ and higher frequency of TNFR2^+^ tregs are associated with inflammatory conditions in type 1 diabetic patients. *Mediators of Inflammation*.

[13] Luopajärvi K, Nieminen J, Ilonen J, Åkerblom HK, Knip M, Vaarala O (2012). Expansion of CD4^+^CD25^+^FOXP3^+^ regulatory T cells in infants of mothers with type 1 diabetes. *Pediatric Diabetes*.

[14] Ryba M, Malinowska E, Rybarczyk-Kapturska K, Brandt A, Myśliwiec M, Myśliwska J (2011). The association of the IVS1-397T>C estrogen receptor *α* polymorphism with the regulatory conditions in longstanding type 1 diabetic girls. *Molecular Immunology*.

[15] Targher G, Bertolini L, Zoppini G, Zenari L, Falezza G (2005). Increased plasma markers of inflammation and endothelial dysfunction and their association with microvascular complications in Type 1 diabetic patients without clinically manifest macroangiopathy. *Diabetic Medicine*.

[16] Goldberg RB (2009). Cytokine and cytokine-like inflammation markers, endothelial dysfunction, and imbalanced coagulation in development of diabetes and its complications. *The Journal of Clinical Endocrinology and Metabolism*.

[17] Lim AK, Tesch GH (2012). Inflammation in diabetic nephropathy. *Mediators of Inflammation*.

[18] Navarro-González JF, Mora-Fernández C (2008). The role of inflammatory cytokines in diabetic nephropathy. *Journal of the American Society of Nephrology*.

[19] Myśliwiec M, Balcerska A, Zorena K, Myśliwska J, Lipowski P, Raczyńska K (2008). The role of vascular endothelial growth factor, tumor necrosis factor alpha and interleukin-6 in pathogenesis of diabetic retinopathy. *Diabetes Research and Clinical Practice*.

[20] Aiello LP, Wong J-S (2000). Role of vascular endothelial growth factor in diabetic vascular complications. *Kidney International*.

[21] Schrijvers BF, Flyvbjerg A, de Vriese AS (2004). The role of vascular endothelial growth factor (VEGF) in renal pathophysiology. *Kidney International*.

[22] Ryba-Stanisławowska M,  Skrzypkowska M, Myśliwska J, Myśliwiec M (2013). The serum IL-6 profile and Treg/Th17 peripheral cell populations in patients with type 1 diabetes. *Mediators of Inflammation*.

[23] Kimura A, Kishimoto T (2010). IL-6: regulator of Treg/Th17 balance. *European Journal of Immunology*.

[24] Cook A (2006). Th17 cells in inflammatory conditions. *The Review of Diabetic Studies*.

[25] Bradshaw EM, Raddassi K, Elyaman W (2009). Monocytes from patients with type 1 diabetes spontaneously secrete proinflammatory cytokines inducing Th17 cells. *The Journal of Immunology*.

[26] Honkanen J, Nieminen JK, Gao R (2010). IL-17 immunity in human type 1 diabetes. *The Journal of Immunology*.

[27] Ryba-Stanisławowska M,  Skrzypkowska M, Myśliwiec M, Myśliwska J (2013). Loss of the balance between CD4^+^Foxp3^+^ regulatory T cells and CD4^+^IL17A^+^ Th17 cells in patients with type 1 diabetes. *Human Immunology*.

[28] American Diabetes Association (2008). Diagnosis and classification of diabetes mellitus. *Diabetes Care*.

[29] Myśliwska J, Rutkowska A, Hak Ł, Siebert J, Szyndler K, Rachoń D (2009). Inflammatory response of coronary artery disease postmenopausal women is associated with the *IVS1-397T* > C estrogen receptor *α* polymorphism. *Clinical Immunology*.

[30] Herrington DM, Howard TD, Bridget Brosnihan K (2002). Common estrogen receptor polymorphism augments effects of hormone replacement therapy on E-selectin but not C-reactive protein. *Circulation*.

[31] Herrington DM, Howard TD, Hawkins GA (2002). Estrogen-receptor polymorphisms and effects of estrogen replacement on high-density lipoprotein cholesterol in women with coronary disease. *The New England Journal of Medicine*.

[32] Sukovich DA, Kauser K, Shirley FD, DelVecchio V, Halks-Miller M, Rubanyi GM (1998). Expression of interleukin-6 in atherosclerotic lesions of male ApoE- knockout mice: inhibition by 17beta-estradiol. *Arteriosclerosis, Thrombosis, and Vascular Biology*.

[33] Pfeilschifter J, Köditz R, Pfohl M, Schatz H (2002). Changes in proinflammatory cytokine activity after menopause. *Endocrine Reviews*.

[34] Rachoń D, Suchecka-Rachoń K, Hak Ł, Myśliwska J (2006). Effects of intranasal 17beta-estradiol administration on serum bioactive interleukin-6 and C-reactive protein levels in healthy postmenopausal women. *Menopause*.

[35] Schalkwijk CG, Stehouwer CDA (2005). Vascular complications in diabetes mellitus: the role of endothelial dysfunction. *Clinical Science*.

[36] Benjamin LE (2001). Glucose, VEGF-A, and diabetic complications. *The American Journal of Pathology*.

[37] Ferrara N, Gerber H-P, LeCouter J (2003). The biology of VEGF and its receptors. *Nature Medicine*.

[38] Yoo SA, Bae DG, Ryoo JW (2005). Arginine-rich anti-vascular endothelial growth factor (anti-VEGF) hexapeptide inhibits collagen-induced arthritis and VEGF-stimulated productions of TNF-alpha and IL-6 by human monocytes. *The Journal of Immunology*.

[39] Verthelyi D (2001). Sex hormones as immunomodulators in health and disease. *International Immunopharmacology*.

[40] Shelbaya S, Amer H, Seddik S (2012). Study of the role of interleukin-6 and highly sensitive C-reactive protein in diabetic nephropathy in type 1 diabetic patients. *European Review for Medical and Pharmacological Sciences*.

[41] Huang Z, Yang B, Shi Y (2012). Anti-TNF-*α* therapy improves Treg and suppresses Teff in patients with rheumatoid arthritis. *Cellular Immunology*.

[42] Miossec P (2009). IL-17 and Th17 cells in human inflammatory diseases. *Microbes and Infection*.

[43] Robak E, Kulczycka-Siennicka L, Gerlicz Z, Kierstan M, Korycka-Wolowiec A, Sysa-Jedrzejowska A (2013). Correlations between concentrations of interleukin (IL)-17A, IL-17B and IL-17F, and endothelial cells and proangiogenic cytokines in systemic lupus erythematosus patients. *European Cytokine Network*.

[44] Chung AS, Wu X, Zhuang G (2013). An interleukin-17-mediated paracrine network promotes tumor resistance to anti-angiogenic therapy. *Nature Medicine*.

